# Neural Underpinnings of Obesity: The Role of Oxidative Stress and Inflammation in the Brain

**DOI:** 10.3390/antiox9101018

**Published:** 2020-10-20

**Authors:** Caitlyn A. Mullins, Ritchel B. Gannaban, Md Shahjalal Khan, Harsh Shah, Md Abu B. Siddik, Vijay K. Hegde, P. Hemachandra Reddy, Andrew C. Shin

**Affiliations:** 1 Neurobiology of Nutrition Laboratory, Department of Nutritional Sciences, College of Human Sciences, Texas Tech University, Lubbock, TX 79409, USA; caitlyn.mullins@ttu.edu (C.A.M.); ritchel-bueno.gannaban@ttu.edu (R.B.G.); harsh.shah@ttu.edu (H.S.); 2 Obesity and Metabolic Health Laboratory, Department of Nutritional Sciences, College of Human Sciences, Texas Tech University, Lubbock, TX 79409, USA; md-shahjalal.khan@ttu.edu (M.S.K.); bakkar.siddik.siddik@ttu.edu (M.A.B.S.); vijay.hegde@ttu.edu (V.K.H.); 3 Department of Internal Medicine, School of Medicine, Texas Tech University Health Sciences Center, Lubbock, TX 79409, USA; hemachandra.reddy@ttuhsc.edu

**Keywords:** antioxidants, anti-inflammatory agents, hypothalamus, hippocampus, prefrontal cortex, ER stress, mitochondrial dysfunction

## Abstract

Obesity prevalence is increasing at an unprecedented rate throughout the world, and is a strong risk factor for metabolic, cardiovascular, and neurological/neurodegenerative disorders. While low-grade systemic inflammation triggered primarily by adipose tissue dysfunction is closely linked to obesity, inflammation is also observed in the brain or the central nervous system (CNS). Considering that the hypothalamus, a classical homeostatic center, and other higher cortical areas (e.g. prefrontal cortex, dorsal striatum, hippocampus, etc.) also actively participate in regulating energy homeostasis by engaging in inhibitory control, reward calculation, and memory retrieval, understanding the role of CNS oxidative stress and inflammation in obesity and their underlying mechanisms would greatly help develop novel therapeutic interventions to correct obesity and related comorbidities. Here we review accumulating evidence for the association between ER stress and mitochondrial dysfunction, the main culprits responsible for oxidative stress and inflammation in various brain regions, and energy imbalance that leads to the development of obesity. Potential beneficial effects of natural antioxidant and anti-inflammatory compounds on CNS health and obesity are also discussed.

## 1. Introduction

Obesity is considered today as a global health problem. Its prevalence stands at over 40% in the US alone, affecting more than 90 million adults and nearly 14 million children [1,2]. Moreover, obesity is recognized as a strong risk factor for serious health conditions, such as cardiovascular disease, Type 2 diabetes, and cancer, all of which are leading causes of mortality in the US and around the world [3]. Decades of research dedicated to understanding the pathogenesis of obesity has clearly demonstrated that developing effective strategies to treat/prevent obesity is difficult due to its multi-etiological nature. Systemic inflammation, mainly characterized by elevated circulating pro-inflammatory cytokines, is considered as one of the major dysregulated conditions that is tightly related to obesity and diabetes [4]. While it represents a collection of typically short-lived, defensive biological responses to infections and other insults that are designed to sustain and recover the health of an organism, the detrimental role of chronic low-grade inflammatory process in the development of obesity has been well appreciated in the last 25 years. Obesity-associated inflammation is also causally linked to a host of metabolic changes, such as insulin resistance and impaired glucose metabolism that disrupt energy homeostasis [5], thus raising the urgency to gain a better understanding of the source(s) of inflammation and the underlying cellular/molecular mechanisms that initiate this inflammatory process.

Local inflammation that occurs in tissues including white adipose tissue (WAT), liver, and muscle is ultimately responsible for increasing circulating pro-inflammatory cytokines and inducing systemic inflammation in obesity [6]. A large body of evidence suggests that the initial inflammatory process is triggered by increased oxidative stress through impaired cellular functions such as endoplasmic reticulum (ER) stress and mitochondrial dysfunction [7]. While mitochondria play a pivotal role in generating cellular energy in the form of ATP and maintaining calcium homeostasis among others [8,9], ER is essential for proper protein synthesis, calcium storage, and lipid metabolism [10]. In the presence of obesity, excess nutrients place tremendous stress on the TCA cycle and the electron transport chain (ETC) in the mitochondria [11] and on unfolded protein response (UPR) in the ER which induces accumulation of misfolded proteins, leading to ER stress [12]. This in turn can lead to structural and functional alterations, causing impaired energy metabolism and excess production of reactive oxygen species (ROS) that will eventually induce oxidative stress and inflammation.

Unlike peripheral tissues, inflammation in the brain or the central nervous system (CNS) has received relatively less attention in the field of obesity and related metabolic disorders. It has now become clear that oxidative stress and inflammation also occur in the brain and have the capacity to impair the CNS control of body weight and appetite [13,14,15]. A number of circulating pro-inflammatory cytokines are found to cross the blood–brain barrier (BBB) through a saturable transport mechanism [16] and enter the brain to exert harmful effects potentially on both neurons and supporting glial cells. Recent studies also suggest that various types of nutrients/metabolites and molecules from the gut (e.g., long-chain fatty acids, ceramides, and lipopolysaccharides via leaky gut permeability) can easily reach the brain and induce cellular stress/inflammatory responses, mainly via Toll-like receptor 4 (TLR4) during obesity development [17,18,19,20]. Given that the role of the brain is to integrate a myriad of nutrient-related signals and tightly control energy homeostasis through modulating the autonomic, endocrine, and behavioral effectors, it is reasonable to speculate that inflammation in the CNS may potentially serve as an important contributing factor to energy imbalance. In support of this concept, high-fat diet (HFD) feeding increases brain inflammation in rats [21], and pro-inflammatory genes such as TNF-α and IL-6 are elevated in the mediobasal hypothalamus (MBH), the homeostatic brain center critical for the control of energy balance, long before they are present in liver or WAT [22]. In fact, just one day of HFD exposure is sufficient to induce inflammatory markers in the MBH before any substantial weight gain [22], suggesting that HFD-induced hypothalamic inflammation is probably one of the earliest events that plays a causal role in obesity development. A recent study demonstrating that mice devoid of astrocytic inflammatory capacity are resistant to diet-induced obesity with improved glucose handling [23] is aligned with this idea, and further underscores the need to better understand the origin(s) and underlying mechanisms of CNS inflammation to fight obesity.

In this review, we provide an overview of the association between CNS oxidative stress/inflammation and obesity. In particular, mitochondrial dysfunction and ER stress—primary defects responsible for production and accumulation of reactive oxygen species (ROS) or free radicals—in the hypothalamus as well as other brain areas involved in processing reward, emotion, and executive functions are separately described. We also discuss current evidence on the roles of antioxidants and anti-inflammatory agents in obesity-related CNS oxidative stress and inflammation, and their potential uses to correct obesity.

## 2. Mitochondrial Dysfunction in the CNS

### 2.1. Hypothalamus

Widely recognized as a classical homeostatic center, the hypothalamus located at the base of the brain comprises numerous nuclei including the arcuate nucleus, ventromedial nucleus, dorsomedial nucleus, and paraventricular nucleus, all of which collectively regulate energy homeostasis [24]. The hypothalamus serves as an integrative energy sensor by detecting a number of nutrients and related hormonal signals from the periphery, and integrating this information with other brain inputs in order to express highly orchestrated responses via endocrine, autonomic, and behavior effectors [25].

Adequate energy production from mitochondria in neurons (as with other cell types) is crucial for optimal signal transmission within the brain and to peripheral organs in order to maintain energy homeostasis [26], so it is not surprising to find hypothalamic mitochondrial dysfunction in obesity. Among other important proteins, mitofusin 2 (Mfn2) is a GTPase located in the outer membrane of the mitochondria, and it plays a vital role in mitochondrial fusion that enables sharing essential components within the mitochondrial population [27]. Carraro and colleagues have recently shown that Mfn2 is substantially decreased in the hypothalamus of HFD-induced obese mice [28]. This may reflect an early, initial stage in obesity development because just 4 days of HFD feeding in the absence of significant weight gain was enough to downregulate hypothalamic Mfn2 mRNA [29]. Importantly, virus-mediated overexpression of Mfn2 in the arcuate nucleus of diet-induced obese mice effectively reduced body weight, adiposity, and food intake, indicating that hypothalamic Mfn2 underlies metabolic alterations and thus may be a critical element in the central regulation of energy balance [29]. In regards to potential underlying mechanisms, it is conceivable that the reduced Mfn2 expression results in unhealthy mitochondria that would ultimately lead to impaired neuronal signaling/functions and communication.

The melanocortin system in the hypothalamus during obesity appears to be directly affected by mitochondrial dysfunction. It consists of several key neuronal populations localized in the arcuate nucleus, namely, agouti-related protein (AgRP)/neuropeptide Y (NPY), and proopiomelanocortin (POMC)/cocaine- and amphetamine-regulated transcript(CART) neurons [24]. These neurons are able to sense nutrients and related hormones such as leptin and insulin depending on the nutritional status. During the fed state, α-melanocyte-stimulating hormone (α-MSH) is released from POMC/CART-expressing neurons that bind to melanocortin receptor 3 and 4 (MC3/4R) to suppress feeding and increase energy expenditure, whereas upon fasting, neuropeptides AgRP and gamma aminobutyric acid (GABA) are released from AgRP/NPY-expressing neurons to bind to MC4R and inhibit POMC neurons, respectively, thereby stimulating feeding and lowering energy expenditure [24,30]. Considering their pivotal role in the control of energy balance, the failure of mitochondrial function in these neurons is expected to disrupt neuronal processing and transmission so as to likely promote appetite and weight gain. After first observing that Mfn2 expression was significantly and consistently decreased in the hypothalamus of mice in response to HF feeding from 4 days up to as long as 12 weeks, Schneeberger and colleagues [29] demonstrated that Mfn2 deletion selectively in POMC neurons caused severe obesity that was accompanied by impaired post-translational POMC cleavage into α-MSH. At the cellular level, the lack of Mfn2 in POMC neurons decreased the activity of Complex I of the ETC and elevated ROS production leading to ER stress, suggesting that impaired mitochondrial fusion may promote a series of events conducive to interference of the melanocortin system and induction of oxidative stress, resulting in obesity. On the other hand, mice lacking Mfn2 specifically in AgRP neurons do not display any morphological or molecular signs of ER stress or inflammation in the hypothalamus, and they are surprisingly resistant to HFD-induced obesity [31]. Interestingly, they stay leaner compared to HFD-fed wildtype controls in spite of lowered POMC expression. While the role of mitochondrial fusion in POMC vs. AgRP neurons seems to differ, both studies described here support a potential causal link between neuronal mitochondrial dynamics within the CNS melanocortin system and energy balance.

Consistent with the findings in POMC neurons, defects within the hypothalamic mitochondrial biogenesis have been reported in the obese state. Colombani and colleagues [32] showed that genetically obese Zucker rats display increased hypothalamic ROS content in response to a low glucose load. While mitochondria generally produce a high amount of ROS during the process of oxidative phosphorylation, key neutralizing enzymes such as superoxide dismutase (SOD) and glutathione peroxidase (GPx) ensure that a low mitochondrial ROS level is maintained. Unfortunately, both SOD and GPx were found to be significantly decreased in the hypothalamus of obese Zucker rats and this most probably explains the increased ROS levels. These findings are also supported by more recent work by Kovačević and colleagues, which demonstrated that a fructose-enriched diet combined with variable stressors in female rats renders obesity and dramatically lowers hypothalamic antioxidant enzymes such as SOD, glutathione reductase, and catalase [33]. Specific defects within the ETC of the mitochondria also have been observed in diet-induced obesity. Ten weeks of HFD feeding in male Swiss mice resulted in increased adiposity that is associated with oxidative damage markers in the hypothalamus, including malondialdehyde and carbonylated proteins, likely due to reduced antioxidant markers such as glutathione [34,35]. Importantly, the activity of Complex I, II, and IV within the hypothalamic mitochondrial ETC was significantly impaired in the obese mice. These failing mitochondrial capacities may well contribute to induction of inflammation leading to obesity. On the other hand, a recent study indicates that hypothalamic inflammation in fact may precede mitochondrial dysfunction in diet-induced obesity [28]. The study showed that when male Swiss mice are placed on a HFD for 7 days, the inflammatory chemokine fractalkine in the hypothalamus appears first just 3 h after exposure to HFD, followed by a significant downregulation of Mfn2 24 h post-HFD. Treating these mice with infliximab, a monoclonal antibody capable of neutralizing TNF-α, was able to restore hypothalamic Mfn2 protein levels. It is possible that hypothalamic inflammation caused by excess nutrients can disrupt proper mitochondrial functions by first activating TLR signaling pathway. This would then suppress TCA cycle activity and drive the synthesis of microRNAs that target ETC complexes [36,37,38,39]. Altogether, these studies provide strong evidence that multiple defects within the mitochondria—impaired fusion, reduced ROS-neutralizing antioxidants, and compromised oxidative phosphorylation—may be mechanistically linked to the development of obesity and related metabolic perturbations.

### 2.2. Extra-Hypothalamic Areas

Multiple brain regions responsible for emotion, reward, and executive functions are known to be intimately linked to hypothalamic regulation of body weight and feeding [40]. This raises the possibility that neuronal defects caused by abnormal mitochondrial functions in these brain areas may send inappropriate signals to the hypothalamus, consequently altering or overriding the allocated hypothalamic responses to nutrient-related inputs to promote weight gain.

The prefrontal cortex is important for executing inhibitory control (e.g., resisting strong appetite impulse), and neural responses in this area are significantly attenuated in obese individuals [41,42]. Cavaliere and colleagues [43] have revealed that inflammatory and oxidative stress markers such as TNF-α, IL-1β, and malondialdehyde were significantly elevated in synaptosomes in the cortex of obese mice fed a HFD for 18 weeks compared to those in a chow-fed lean control group. Antioxidant glutathione (GSH) was found to be markedly reduced, and the ratio of GSH to its oxidized form GSSG, a readout for antioxidant activity, was decreased, as expected in diet-induced obese mice. Consistent with this, free radical scavengers such as SOD and aconitase were significantly decreased, which most likely exerted a negative impact on mitochondrial function as evidenced by a significant reduction in mitochondrial state 3 respiration in maximal respiration capacity. These results are in agreement with the increased superoxide production and swelling—an indicator of permeability transition pore—selectively in the mitochondria within the cortex of mice fed a HFD for 16 weeks [44]. Although these studies do not establish a clear link between mitochondrial defects specifically in the prefrontal cortex and obesity, other investigators have demonstrated similar results in this specific brain area. Swiss mice rendered obese after exposure to 10–13 weeks of HFD or s similar energy-dense cafeteria diet displayed suppressed activity of citrate synthase and isocitrate dehydrogenase, enzymes responsible for catalyzing critical reactions in the mitochondrial TCA cycle, and impaired Complex II activity in the prefrontal cortex [35,45,46].

The hippocampus is an essential brain region in the limbic system that governs learning and memory. Our appetitive and consummatory behavior is determined not only by monitoring the energy availability in the body and the detection of hormonal signals, but also through our knowledge and reward/hedonic expectancy of foods based on its quality and related contextual cues [47]. Importantly, much of this information is processed and retrieved from our memory. Thus, it has become clear that the hippocampus actively participates in the decision-making of food consumption by altering the prediction of hedonic consequences of feeding [47]. Neuroimaging studies have shown that hippocampal volume is diminished with aberrant neural activity in obese individuals compared to lean healthy individuals [48,49,50], indicating a potential mechanistic link between hippocampal function and metabolic health. Consistent with these results, rodent studies indicate that diet-induced obesity (via HFD or high-sucrose diet) manifests impaired memory consolidation [51,52,53] that is associated with hippocampal BBB disruption [54,55], thereby potentially increasing the entry of systemic inflammatory mediators into the hippocampus. Indeed, diet-induced obese animals exhibit microglial activation, increased production of ROS, and pro-inflammatory cytokines including IL-1β and TNF-α in the hippocampus [54,55]. As shown in the prefrontal cortex, HFD feeding is likely capable of compromising ATP synthesis in the hippocampus through lowering the activity of citrate synthase in mitochondrial TCA cycle and suppressing the activity of Complex I, II, and IV [35,46].

It is only recent that the mitochondrial defects in other brain regions have started to receive more attention. The striatum lies in the subcortical basal ganglia and regulates reward processes and motivation. Dysfunctional dopamine and other neural signaling in this particular brain structure have been hypothesized as some of the major contributors to overeating and the development of obesity [56,57,58,59,60]. It is thus conceivable that the striatum is quite susceptible to inflammatory insult and mitochondrial stress. Mice rendered obese by 10 weeks of HFD feeding display elevated inflammatory mediators such as TNF-α and IL-1β in the striatum and the corresponding oxidative damage, as evidenced by increased carbonylated proteins and lower glutathione [35]. Notably, the activity of ETC Complex I, II, and IV was significantly reduced in the striatum of these mice compared to that in lean healthy mice. Consistent with these results, a more recent study by de Farias and colleagues has shown that 11 weeks of HFD feeding induces a significant weight and fat gain, and these are associated with an impaired mitochondrial respiratory chain in the striatum [46].

While the underlying mechanisms for CNS mitochondrial dysfunction in diet-induced obesity are not clear, current research points to a potential glitch in post-translational modification. NAD-dependent deacetylase sirtuin-3 (SIRT3), a soluble protein located in the mitochondrial matrix, plays a key role in vital metabolic processes including fatty acid oxidation, oxidative phosphorylation, and antioxidant defense via deacetylating mitochondrial enzymes under stress [61]. Interestingly, SIRT3 mRNA in the brain was found to be low in diet-induced obese mice [62]. Further establishing the link between SIRT3, mitochondria, and obesity, mice with SIRT3 deletion while exposed to a HFD displayed weight gain and brain protein hyperacetylation, microglial activation, neuroinflammation, and defective mitochondrial respiration that are more pronounced than those in HFD-fed obese wildtype (WT) mice [63]. Cyclophilin D (CyPD) is a chaperone protein regulated by SIRT3, and is essential for controlling the mitochondrial permeability transition pore (MPTP), opening of which results in impaired ATP synthesis and elevated ROS production [64]. Whether or not CypD expression in the brain is elevated with a corresponding reduction in SIRT3 during obesity is unknown. It is speculated that by deacetylating and inactivating CyPD, SIRT3 may be able to protect neurons from oxidative stress by inhibiting MPTP formation that is conducive to increased ROS production and apoptosis, thereby help maintain optimal neuronal functions and energy homeostasis. In support of this concept, Devalaraja-Narashimha and colleagues have demonstrated that a global CyPD knockout (KO) confers resistance to diet-induced obesity in both male and female mice most likely via an increased energy expenditure [65]. Brain-specific or region-specific CyPD KO would be necessary to dissect its role in regulating mitochondrial function and energy balance.

## 3. Endoplasmic Reticulum (ER) Stress in the CNS

The ER is a large membrane-enclosed organelle that plays an important role in protein folding and maturation, calcium homeostasis, and lipid metabolism [66]. When faced with various cellular stressors, such as altered redox balance, hypoxia, and nutrient deprivation, the unfolded-protein response (UPR) program is activated to alleviate the burden of misfolded proteins by enhancing protein folding and degradation capacity [67]. A hyper-stimulation by any of these biological insults, however, would saturate the capacity and eventually lead to ER stress, or the inability of ER to properly fold proteins. ER stress is an important contributing factor for the development of obesity and related diseases including type 2 diabetes, cardiovascular disease, and neurodegenerative disorders [68,69]. Moreover, obesity-induced ER stress and the resultant inflammatory processes are causally linked to insulin resistance and dysregulated glucose metabolism in peripheral tissues, including liver, adipose tissue, and pancreas [70,71,72]. Here we discuss some of the research over the last 15 years that has bridged the gap of knowledge between ER stress in the brain and obesity.

### 3.1. Hypothalamus

Both in vivo and in vitro studies have demonstrated a close association between excess nutrition (especially high-fat) and hypothalamic ER stress. Mice exposed to HFD for 8 weeks developed obesity and increased hypothalamic pPERK, a phosphorylation-activated enzyme that attenuates protein synthesis in response to ER stress [13]. Ozcan and colleagues [73] have reported that 20 weeks of HFD feeding raises pPERK and pIRE1, an ER transmembrane RNAse that activates inflammatory and apoptosis pathways in response to ER stress, in the hypothalamus of mice. Similar results were observed in diet-induced obese rats in which HFD intake in Wistar rats for 8 weeks increased ER stress and stimulated microglial activation and pro-inflammatory cytokines like IL-6, TNF-α, and IL-1β in the hypothalamus [18]. Interestingly, Sprague Dawley (SD) rats fed a HFD for 12 weeks displayed increased adiposity that was associated with elevated pPERK and phosphorylation of eIF2α (critical initiation factor downstream of PERK) specifically in the arcuate nucleus of the hypothalamus, indicating a possible detrimental impact on the arcuate melanocortin system that consists of orexigenic NPY/AgRP and anorexigenic POMC/CART neurons [74]. A direct effect of lipids on ER stress and inflammation in the brain was demonstrated by Contreras and colleagues [75] in which an intracerebroventricular (ICV) infusion of ceramide in SD rats for 5 days significantly increased ER stress and inflammatory markers. Likewise, an ICV treatment with arachidonic acid, a long-chain fatty acid, for 3 days induced both hypothalamic ER stress and inflammation, as shown by increased pPERK, peIF2α, and TLR4 protein expression in rats [18]. Of note, this occurred as early as at treatment day 1 when body weight was not different between groups, suggesting that lipid content in HFD per se can directly affect hypothalamic ER stress independent of obesity development. This is supported by an in vitro study showing that 12h treatment with palmitic acid (saturated fatty acid), but not palmitoleic acid (monounsaturated fatty acid), stimulated ER stress responses, including GRP78, an ER stress sensor, and increased ROS production in mHypoA-CLU472 hypothalamic cell line [76].

Leptin and insulin are two major hormones that convey signals related to energy availability to the brain to regulate energy balance [77]. CNS leptin and insulin resistance are linked to obesity and related metabolic disorders, and several studies suggest that ER stress may be causally associated with this dysregulation. Won and colleagues [78] have shown that a single ICV injection of thapsigargin, an ER stress inducer, in mice increases hypothalamic ER stress markers such as pIRE1 and CHOP as expected, and reduces leptin’s appetite-suppressing action. This is likely due to hypothalamic leptin resistance as evidenced by lower STAT3 activation in response to leptin. Similarly, thapsigargin administration directly in the hypothalamus inhibited the ability of insulin to suppress food intake, and this was accompanied by lower hypothalamic phosphorylation of Akt, a key protein in insulin signaling pathway [78]. Consistent with these findings, mice with neuron-specific deletion of XBP1, a transcription factor critical for adapting to ER stress, increased pPERK in the hypothalamus and developed severe obesity when challenged with HFD [73]. This was associated with leptin resistance, significantly higher food intake, and lower energy expenditure.

Others have investigated hypothalamic ER stress as a major driver for the maintenance of obesity. While the ICV ceramide infusion in mice significantly increased food intake and body weight and hypothalamic ER stress markers, GRP78 overexpression in the ventromedial nucleus of the hypothalamus was able to reverse diet-induced obesity by enhancing brown adipose thermogenesis [75]. The same group recently showed that hypothalamic overexpression of GRP78 in HFD-fed obese mice is sufficient to alleviate ER stress in the hypothalamus and abolish the obese and metabolic phenotype [79]. The UPR-induced ER chaperones, including GRP78, are necessary to reduce ER stress by lowering the aggregation of misfolded proteins [80]. A chronic treatment with chemical chaperones such as 4-phenyl butyrate (PBA) and tauroursodeoxycholic acid (TUDCA) in either genetically obese *ob/ob* mice or HFD-induced obese mice was shown to significantly lower body weight, food intake, and hypothalamic pPERK, and increase leptin sensitivity and energy expenditure [73], suggesting that alleviation of ER stress by chaperones may be directly responsible for reversing the obese phenotype. In agreement with the role of ER stress in obesity, Williams and colleagues [81] have demonstrated that an inducible, constitutive expression of XBP1 selectively in hypothalamic POMC neurons of mice placed on a HFD can confer resistance to diet-induced obesity, most likely due to enhanced leptin and insulin sensitivity and increased energy expenditure. This raises the possibility that POMC neurons in the hypothalamus may be sensitive to HFD and the related ER stress. Indeed, 8 months of HFD feeding resulted in a 25% reduction in the number of POMC neurons in obese mice, and Hsp72, a chaperone protein expressed in response to cellular injury, was noticeably elevated in POMC neurons only after 7 days of HFD exposure [22]. POMC neuronal damage during diet-induced obesity is further supported by Cakir and colleagues, whose findings showed that HFD exposure for 12 weeks in SD rats induces ER stress in the arcuate nucleus and disrupts post-translational processing of POMC [74]. This was indicated by a dramatically decreased cleaving enzyme PC2, leading to lower levels of α-MSH, a cleaved product of POMC. Induction of ER stress by an ICV injection of either thapsigargin or tunicamycin in chow-fed lean rats recapitulated the impaired POMC processing observed in diet-induced obese rats. Importantly, treating these animals with a chemical chaperone TUDCA for 2 days led to a significant decrease in body weight, food intake, and ER stress in the arcuate nucleus that is associated with restored arcuate α-MSH levels. Preservation of PC2 protein with a chemical chaperone PBA or salubrinal (inhibitor of ER stress) treatment in tunicamycin-treated hypothalamus cell line supports the concept that proper ER functionality is vital for POMC neuronal functions and the control of energy balance.

What could be the potential underlying mechanisms that trigger hypothalamic ER stress in obesity? Several studies point to glial cells, the non-neuronal cells (e.g., astrocytes, microglia) that assist and protect neurons. Both cell types are distributed throughout the brain, including the hypothalamus. While astrocytes are shown to be involved in modulating metabolic signals in the melanocortin system through their ability to detect glucose and fatty acids [82], hypothalamic microglia are activated and undergo functional and morphological changes when flooded with dietary fat [83]. Excessive stimulation of these supporting cells may lead to gliosis or glial activation, and induce ER stress and local inflammation in the hypothalamus, which are causally linked with obesity development [83]. Baufeld and colleagues [84] have recently shown that compared to regular chow-fed mice, those on a HFD for 8 weeks significantly increased their body weight and induced hypothalamic gliosis, as evidenced by a greater immunoreactivity for Iba1 and GFAP, a microglial and astrocyte marker, respectively. An increased number of Iba1-positive cells was also observed specifically in the hypothalamus of postmortem obese individuals (BMI > 30) compared to those of lean individuals. Interestingly, hypothalamic microglial markers (Cd68, Emr1), microglial accumulation, and pro-inflammatory cytokines were all found to be increased in rats as early as 3 days after HFD consumption before any significant gain in adiposity [22]. These findings suggest that activation of microglia may be one of the early events that trigger obesity development. Consistent with these results, a recent study [85] demonstrated that activating microglia by deletion of A20 (an anti-inflammatory molecule and a negative regulator of NF-kB) in mice results in hypothalamic microglia recruitment, neuroinflammation, and a significant weight gain. Along with the observation [86] that mice fed with a diet specifically high in saturated fatty acids (stearic, lauric, and palmitic acid) display microglial activation and inflammation in the hypothalamus, these findings collectively suggest that HFD rich in saturated fatty acids may initiate the development of obesity mainly through stimulation of hypothalamic gliosis and the resultant ER stress and neuroinflammation.

### 3.2. Extra-Hypothalamic Areas

Unlike in the hypothalamus, ER stress in other brain regions has not been extensively explored yet. Diet-induced obese mice following 20 weeks of HFD displayed higher expression of hippocampal ER markers (peIF2α and pPERK), and inflammatory markers such as CD11b, GFAP, TNF-α, and IL-2 [87]. This was associated with impaired insulin signaling in the hippocampus, as indicated by lower pIRS1, pAKT, PI3K, pmTOR, and phosphorylation of S6 kinase. ER stress may be linked to obesity per se and not depend on the composition of HFD since genetically obese *db/db* mice on a regular chow diet present a significant downregulation of many chaperones in the hippocampus that are critical to alleviate ER stress [88]. Similarly, Sims-Robinson and colleagues have shown that hippocampal ER stress is induced in HFD-fed obese mice and regular chow-fed ApoE/ApoB/ob triple KO (ApoE mice), but not in LDLR/ApoB/ob triple KO (LDLR mice) [89]. Both ApoE and LDLR mice are known to be obese and hyperlipidemic [90]. Interestingly, only diet-induced obese mice and ApoE mice display hyperglycemia and insulin resistance, suggesting that these defects may be associated with hippocampal ER stress. The ability of ER stress to activate the inflammatory c-Jun N-terminal kinase (JNK) pathway and impair insulin signaling via serine phosphorylation of IRS1 [89,91] supports a potential mechanistic link between ER stress and insulin resistance in the hippocampus. Of note, HFD-fed obese SD rats increased markers of hippocampal ER stress and apoptosis that were completely normalized along with body weight following 8 weeks of treadmill exercise [92], suggesting that obesity and associated ER stress in the hippocampus can be largely reversed by paring with a simple exercise intervention.

The amygdala is a part of the limbic system involved in emotional behavior and feeding [93]. Decreased connectivity between the amygdala and areas related to learning and emotion such as the hippocampus, midbrain, and thalamus is linked to obesity [94], indicating possible malfunctions within the amygdala leading to impaired projections and interactions with other reward and homeostatic regulatory centers in the brain. Castro and colleagues [95] have shown that HFD feeding in Wistar rats for 8 weeks induces a nearly 170% increase in weight gain and elevates ER stress markers (pPERK and IRE1α) and inflammatory markers (pJNK and pIK K) in the amygdala. They further demonstrated that while insulin injection in the amygdala does not activate insulin signaling and reduce food intake in diet-induced obese rats, an oral administration of PBA (inhibitor of ER stress) for 7 days is able to substantially reduce ER stress, food intake, and increase insulin signaling in the amygdala, suggesting that ER stress may precede and contribute to the development of insulin resistance in the amygdala in a setting of nutrient surplus.

Reddy and colleagues [96] have reported increased ER stress markers, such as ATF6, IRE1, and CHOP, in the cortex of WNIN/ob rats, a mutant obese strain that develops spontaneous obesity with insulin and leptin resistance independent of HFD exposure. Likewise, HFD-fed obese rats displayed increased expression of pPERK, peIF2α, caspase-12, CHOP, and Bax/Bcl-2 in the prefrontal cortex that are indicative of ER stress and ER stress-mediated apoptosis [97]. Considering higher expression of fatty acid transport protein 1 (FATP1) in this brain region in diet-induced obese rats, their findings raise the possibility that excess uptake and accumulation of fatty acids in the prefrontal cortex can induce ER stress, inflammation, neuronal dysfunctions, and apoptosis. This would ultimately suppress inhibitory control over HFD, leading to predisposition to weight gain. Similar to the hippocampus [92], the prefrontal cortex appears to be quite responsive to aerobic exercise as evidenced by a complete rescue of prefrontal ER stress by 8 weeks of treadmill exercise in HFD-fed obese rats.

It is worth noting that ER stress in the CNS may be primarily responsible for defective liver metabolism irrespective of energy imbalance. Horwath and colleagues [98] have identified the subfornical organ (SFO), a brain area located at the base of the lateral ventricle, with significantly higher ER stress markers such as p58, CHOP, and XBP1 in mice fed a HFD for 15 weeks which was associated with hepatic steatosis. Interestingly, overexpression of GRP78 (ER chaperone) specifically in the SFO was able to rescue ER stress and hepatic steatosis independent of energy balance. The SFO is a highly vascularized nucleus that lacks the BBB; therefore, it is conceivable that this region is readily exposed to high levels of HFD-induced circulating fatty acids as well as inflammatory mediators that can trigger ER stress [99]. The restoration of liver health without changes in body weight and food intake suggests that ER stress in the SFO is critical for the pathogenesis of hepatic steatosis, and further reinforces the concept of CNS region-dependent uncoupling between energy homeostasis and nutrient metabolism.

## 4. Role of Antioxidants/Anti-Inflammatory Agents in the CNS

Natural anti-inflammatory and antioxidant agents like polyphenols and flavonoids have been heralded today as potential candidates to fight obesity by driving lipolysis, suppressing adipose tissue expansion, and lowering macrophage accumulation and the resultant inflammation [100]. Here we review limited but growing literature to discuss the potential roles of these natural compounds in ameliorating oxidative stress, ER stress, and inflammation in the CNS during obesity.

### 4.1. Quercetin

A flavonoid found in many fruits, vegetables, and grains, quercetin has a wide range of biological functions, including antioxidant, anti-inflammatory, anti-carcinogenic, anti-obesity activity, and neuroprotection [101,102]. Studies have consistently shown fat and oxidative stress-lowering effects of quercetin in obese mice fed a HFD for 8 weeks or longer [103,104,105]. Liang and colleagues [104] have demonstrated that the offspring of HFD-fed obese dams develop obesity and related metabolic perturbations such as insulin resistance and hyperglycemia as expected, but supplementation of quercetin along with HFD in dams dramatically corrects these metabolic dysfunctions, pointing to a powerful, obesity-curbing effect of quercetin even in the presence of gestational HFD exposure. More recent studies indicate its effect in the CNS. Compared to mice fed a HFD for 8 weeks, mice that were supplemented with quercetin displayed a significant reduction in microglial activation (Iba-1, CD11b) and inflammatory mediators such as TNF-α, IL-1β, and MCP-1 in the hypothalamus [106]. Quercetin’s antioxidant action does not seem to be limited to the hypothalamus, as its supplementation to mice on a HFD diet for 13 weeks was able to significantly lower oxidative stress markers such as malondialdehyde, carbonylated proteins, and ROS in the hippocampus [107]. Interestingly, body weight of these mice was higher than those of mice exposed to HFD diet alone while there was no difference in food intake. The anti-inflammatory properties of quercetin were also confirmed in BV2 microglial cell line and primary microglia isolated from a mouse hypothalamus, indicating its ability to directly suppress microglia activation [106].

### 4.2. Curcumin

Curcumin is the main bioactive ingredient in turmeric (*Curcuma longa*) that has shown antioxidant and anti-inflammatory properties in in-vitro and in-vivo studies [108,109,110,111,112,113,114]. Its ability to reduce or delay cognitive decline in response to aging [115], chronic stress [116], alcohol [117], anti-epileptic drugs [118], and smoking [119] has been reported. Likewise, Wu and colleagues have shown that curcumin treatment in mice with induced ischemic stroke by middle cerebral artery occlusion was able to substantially reduce oxidative and ER stress and infarct size [114]. Franco-Robels and colleagues [120] are one of the first groups to demonstrate a therapeutic antioxidant property of curcumin in the hippocampus in obesity and diabetes. The study showed that compared to genetically obese and diabetic *db/db* control mice, animals treated with curcumin for 8 weeks significantly lowered carbonylated proteins (marker for oxidative damage) in the hippocampus. Supporting these results is the reduction of carbonylated proteins and lipid peroxidation, as measured in the sera of obese male individuals treated with curcumin orally for 12 weeks [120]. A recent study reported that ICR mice rendered obese with 30% fructose solution for 8 weeks display hippocampal microglia activation and neuroinflammation through induction of TLR4/NF-kβ signaling and increased expression of fractalkine, a chemokine mainly produced by microglia, in the hypothalamus [121]. Oral treatment with curcumin for the last 4 weeks was sufficient to resist diet-induced obesity and completely normalize inflammation and microglia activation in both the hippocampus and hypothalamus. Altogether, these studies suggest that curcumin is a natural compound that can potentially lower brain oxidative stress and inflammation to curb obesity.

### 4.3. Resveratrol

Resveratrol is a non-flavonoid polyphenol present in red wine and grape seeds that possesses antioxidant, anti-inflammatory, and neuroprotective functions [122]. It is known to activate nicotinamide adenosine dinucleotide (NAD)-dependent deacetylase (SIRT1), a nuclear protein that mediates beneficial metabolic effects of caloric restriction and lowers pro-inflammatory mediators such as NF-kB and activator protein-1 (AP-1) [123,124]. Resveratrol has been shown to significantly decrease the cytotoxic effects 4-HNE in PC12 cells—a cell line derived from a pheochromocytoma with a neural embryonic origin—by suppressing ROS production [125]. Oral resveratrol supplementation in genetically obese ob/ob mice for 3 weeks lowered lipid peroxidation in the brain as evidenced by decreased malondialdehyde and hydroperoxides [126]. It also enhanced the activities of antioxidant enzymes such as SOD, glutathione peroxidase/reductase, and catalase, however, these beneficial effects were not associated with any changes in body weight. Jeon and colleagues [127] also reported that independent of body weight, HFD-fed mice that are supplemented with resveratrol for 20 weeks exhibit low inflammatory markers (TNF-α, Iba-1) and restored insulin signaling in the hippocampus, along with improved insulin sensitivity and decreased lipid peroxidation in liver. Similar outcomes extend to non-human primates as Rhesus monkeys treated with HFD and resveratrol for 2 years reinstate insulin sensitivity and lower inflammatory response in WAT compared to monkeys with HFD exposure alone, without changes in body weight [128]. Only one study has directly tested the role of resveratrol action in the CNS by chronic ICV infusion of resveratrol in HFD-fed obese mice [129]. As with oral treatment in other studies, central resveratrol was able to attenuate hypothalamic NF-kβ and reverse hyperglycemia and hyperinsulinemia, but body weight and food intake were not affected. Collectively, these findings suggest that resveratrol may be effective in reducing oxidative stress and neuroinflammation that may be causally linked to improved metabolic health.

### 4.4. Celastrol

A compound found in the root extracts of *Tripterygium wilfordii*, celastrol is reported to exhibit antioxidant, anti-inflammatory, anti-cancer, and anti-neurodegenerative activity [130,131]. Saito and colleagues [132] have demonstrated that only 10 days of systemic celastrol injection is sufficient to significantly reduce food intake and body weight in both HFD-fed obese mice and MC4R-deficient obese mice. However, inflammatory mediators including TNF-α and IL-1β were upregulated without any changes in ER stress markers in the hypothalamus of celastrol-treated mice. Another study has shown that either oral or systemic celastrol treatment for 3 weeks in HFD-fed obese mice is able to dramatically lower body weight by suppressing caloric intake and preventing an expected drop in energy expenditure [133]. The corrected body weight can be largely explained by increased leptin sensitivity in the hypothalamus since celastrol was not effective in leptin-deficient *ob/ob* mice or leptin receptor-deficient *db/db* mice. In contrast to the study by Saito and colleagues [132], the anti-obesity action of celastrol was associated with reduced ER stress in the hypothalamus. Kyriakou and colleagues [134] have recently shown that deletion of hypothalamic leptin signaling inhibitors—PTP1B and TCPTP—successfully lowers body weight in diet-induced obese mice, supporting the concept that celastrol restores the control of energy balance through enhancing hypothalamic leptin sensitivity. Whether or not this is linked to lower ER stress or inflammation in the hypothalamus is not clear. Altogether, current literature on the role of celastrol in connecting CNS ER stress/inflammation with body weight regulation is sparse, but the observed effects on energy homeostasis are robust and encouraging.

### 4.5. Epigallocatechin-3-gallate (EGCG)

EGCG is one of the most abundant and potent polyphenols in green tea. As with other polyphenolic compounds, EGCG has been studied for its antioxidant, anti-inflammatory, and anti-obesity effects [135,136,137]. When treated with EGCG for 16 weeks, mice placed on a HFD and 10% fructose solution resisted obesity development and displayed better CNS insulin sensitivity than HFD-fed obese control mice [138]. Further, EGCG attenuated neuroinflammation by lowering TNF-α mRNA and phosphorylation of inflammatory markers p38 and NF-kβ in the brain. Okuda and colleagues [139] have reported similar findings in mice fed with a HFD supplemented with the green tea extract. Mice gained less body weight and fat mass, and this was associated with significantly higher hypothalamic expression of Ikkβ, a kinase known to inhibit NF-kβ activation and inflammation. Not surprisingly, EGCG was able to completely normalize the levels of pro-inflammatory markers such as TNF-α, IL-1β, and IL-6, as well as microglial activation marker Iba-1 in the hypothalamus [140]. Further investigation is warranted to understand if EGCG-led anti-obesity effects are mediated through reduced hypothalamic inflammation.

### 4.6. Grape Seed and Skin Extract (GSSE)

GSSE is a complex polyphenol mixture exerting a wide range of beneficial effects against obesity, dyslipidemia, hypertension, inflammation, neuronal injury, and lipotoxicity [141,142]. Most of these metabolic improvements seem to be attributed to its antioxidant and free radical-scavenging properties [143,144]. Charradi and colleagues [145] have shown that compared to chow-fed Wistar rats, those exposed to a HFD for 6 weeks doubled the weight gain and induced brain lipotoxicity as indicated by elevated levels of malondialdehyde and carbonylated proteins, and lower levels of antioxidant markers including glutathione peroxidase and SOD. However, rats with systemic injection of GSSE during the HFD feeding period were effectively prevented from gaining extra weight, and this was associated with reduced lipotoxicity and increased antioxidant activity in the brain. The same group extended their findings and demonstrated that initiating GSSE supplementation in already HFD-induced obese rats is still effective at conferring resistance to obesity and normalizing lipid peroxidation, protein carbonylation, and H_2_O_2_ in the brain [146]. GSSE-treated animals also displayed an enhanced antioxidant defense system in the brain as evidenced by increased SOD and catalase. Further, this was associated with restored calcium homeostasis and mitochondrial Complex I activity. These findings support a potential role of GSSE in lowering CNS oxidative stress, and future studies using different animal species with more mechanistic approaches will clearly strengthen the link between the anti-obesity effects of GSSE and neuronal health in the brain. The effects of the natural antioxidant and anti-inflammatory compounds are summarized in Table 1.

## 5. Conclusions and Future Perspectives

**Figure 1:**
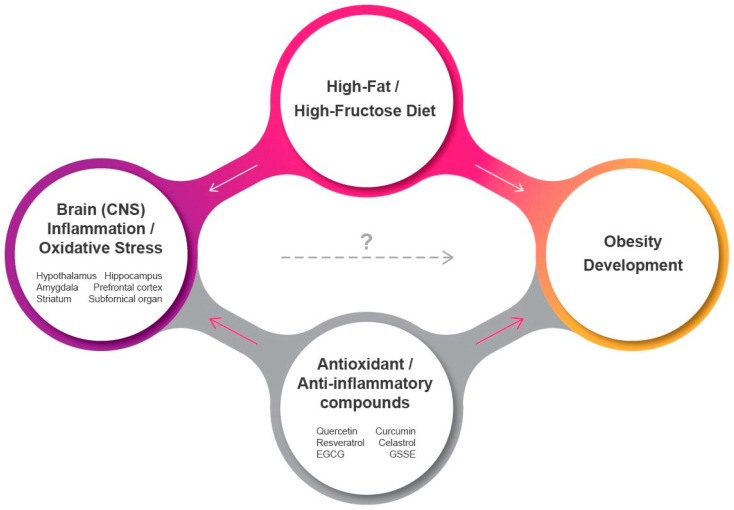
Effects of energy-dense, palatable foods on CNS inflammation and oxidative stress and obesity. White and red arrows indicate stimulatory and inhibitory, respectively. A potential causal link exists between oxidative stress and inflammation in the CNS and induction of obesity following consumption of a high-fat/high-fructose diet. EGCG: epigallocatechin-3-gallate; GSSE: grape seed and skin extract.

Current studies indicate a clear link between oxidative stress/activation of the inflammatory network in the CNS and the pathophysiology of metabolic disorders such as obesity and diabetes. Recognized as the main drivers for ROS-induced cellular damage and neuroinflammation, mitochondrial dysfunction and ER stress in the brain with compromised antioxidant activity are evident in obesity (Figure 1). While the hypothalamus and the melanocortin systemcritical for energy homeostasis are primarily affected by diets rich in high fat and/or high sugar, a number of studies confirm that other brain areas involved in inhibitory control (prefrontal cortex), reward/emotion processing (dorsal striatum, amygdala), and memory (hippocampus) are also quite sensitive to HFD exposure. In addition, a recent study has identified mitochondrial dysfunction and oxidative stress even in the spinal cord where the sympathetic motor neurons originate that control nutrient partitioning and energy expenditure [147]. This has a significant clinical implication as obesity is a strong risk factor for neurological and neurodegenerative diseases in which these specific areas are known to be severely impaired. Thus, central inflammation in obesity not only leads to energy imbalance with higher caloric intake and lower energy expenditure, but can also drive cognitive and motor impairments. Importantly, a growing number of evidence indicate that induction of oxidative stress and inflammation in the CNS may actually precede the development of obesity, suggesting a potential causal link that necessitates detailed mechanistic investigations in regard to various affected brain areas as well as their individual contributions to the extent of obesity. Since the ER and mitochondria depend on one another to coordinate essential cellular functions like calcium signaling, ATP synthesis, and intracellular trafficking [148], understanding how their interorganelle communication becomes aberrant would help gain deeper mechanistic insights. Finally, natural polyphenolic compounds are emerging as promising candidates to fight obesity with their antioxidant and anti-inflammatory properties, although their direct and beneficial role in alleviating CNS-specific oxidative stress and inflammation and its causal link to restored energy balance is not yet convincing and needs to be explored further.

## Figures and Tables

**Table 1:** Effects of natural compounds on energy balance associated with CNS regional oxidative stress and inflammation.

Natural Compounds	Obese Model	Body Weight	Brain Area	Outcomes	Ref.
Quercetin	HFD	NR	Hypothalamus	↓Inflammation	[106]
	HFD	NR	Hippocampus	↓Oxidative stress	[107]
Curcumin	db/db	↓	Hippocampus	↓Oxidative stress	[120]
	30% Fructose	Resisted gain	Hippocampus Hypothalamus	↓Inflammation ↓Microglia activity	[121]
Resveratrol	ob/ob	−	Whole brain	↓Lipid peroxidation ↓Antioxidant activity	[126]
	HFD	−	Hippocampus	↓Inflammation ↓Lipid peroxidation	[127]
	HFD	−	Hypothalamus	↓Inflammation	[129]
Celastrol	MC4R KO	↓	Hypothalamus	−Inflammation −ER stress	[132]
	HFD	↓	Hypothalamus	↓ER stress	[133]
	ob/ob db/db	−			
Epigallocatechin-3-gallate (EGCG)	HFD+ 10% fructose	↓	Whole Brain	↓Inflammation	[138]
	HFD	−	Hypothalamus	↓Inflammation	[139]
Green tea extract (>90% EGCG)	HFD	Resisted gain	Hypothalamus	↓Inflammation	[140]
Grape seed and skin extract (GSSE)	HFD	Resisted gain	Whole brain	↓Lipotoxicityv ↓Antioxidant activity	[145]
	HFD	Resisted gain	Whole brain	↓Lipid peroxidation ↓Oxidative stress ↑Antioxidant activity	[146]

**Abbreviations:** HFD—high-fat diet; MC4R KO—melanocortin 4 receptor knockout; ob/ob—leptin-deficient obese mice; db/db—leptin receptor-deficient obese mice; NR—not reported; no change; **↑**—increased; **↓**—decreased.
